# A deployment-derived online banking fraud detection inference-log dataset from a live cloud-based deep learning system

**DOI:** 10.1016/j.dib.2026.113153

**Published:** 2026-08-08

**Authors:** Hemn Hashim Fatah, Zryan Najat Rashid

**Affiliations:** Technical College of Informatics, Sulaimani Polytechnic University, Sulaymaniyah, Kurdistan Region, Iraq

**Keywords:** Online banking fraud, Deployment-derived dataset, Deep learning, CNN-LSTM, Real-time detection, Class imbalance, Cloud deployment, Threshold calibration

## Abstract

This article describes a dataset of 56,962 transaction inference logs collected through a live, cloud-deployed online banking fraud detection demonstration system over a 31-day collection period from 01 January 2026 to 31 January 2026. The data were gathered through a REST API endpoint hosted on a Ubuntu Linux virtual private server at model-test.online, where a trained hybrid Convolutional Neural Network and Long Short-Term Memory (CNN-LSTM) model processed each incoming transaction in real time. The dataset contains 98 confirmed fraudulent transactions (0.172% fraud rate), reflecting the natural class imbalance encountered in operational environments. Each record comprises 38 columns in total: 30 input features consisting of 28 principal component analysis (PCA)-transformed variables (V1 through V28), the transaction amount (in USD), and a time index; and 8 additional fields including model-generated outputs (fraud probability score, categorical risk level [LOW, MEDIUM, or HIGH], confidence estimate, and action recommendation [BLOCK or ALLOW]), response latency in milliseconds, a UTC timestamp, a partially anonymised IP address, and a transaction identifier. Ground truth fraud labels were assigned through manual verification of model-flagged (BLOCK) transactions during the deployment period; transactions that received an ALLOW recommendation were not individually reviewed and were assigned a legitimate label by default. The deployment was a proof-of-concept system operating as a public demonstration interface and not a licensed banking institution; accordingly, the transactions represent API submissions by the public and the authors during testing, sharing the PCA-transformed feature structure of the ULB Credit Card Fraud Detection benchmark. The dataset is freely available on Zenodo (DOI: 10.5281/zenodo.20030064, CC BY 4.0) to support reproducibility of the accompanying research, to provide a recent deployment-derived fraud benchmark, and to facilitate independent evaluation of fraud detection methods under realistic class imbalance conditions.

Specifications Table

**Table:** 

Subject	Computer Sciences
Specific subject area	Online banking fraud detection using cloud-deployed deep learning models
Type of data	Tabular data — CSV format 56,962 rows × 38 columns (30 input features + 8 additional fields) File name: fraud_tests_export_20260501_080333.csv (21.2 MB)
Data collection	Automatically logged by a REST API endpoint (POST /predict) at each transaction inference call on the live deployment server. Collection window: 01 January 2026 – 31 January 2026 (31 days). Fraud label assignment: at the end of the collection period, all transactions with a BLOCK recommendation (fraud_probability > 0.43) were manually reviewed by the authors; confirmed fraud cases were assigned is_fraud = 1. All ALLOW-recommended transactions were assigned is_fraud = 0 without individual review. This procedure introduces verification bias: any fraud missed by the model threshold is permanently recorded as legitimate. Users should account for this dependency when applying is_fraud as ground truth. Instruments: TensorFlow 2.13.0 CNN-LSTM model, Flask REST API, MySQL 8.0 database, Ubuntu 24.04 LTS VPS.
Data source location	Sulaymaniyah, Kurdistan Region, Iraq. Server: model-test.online (Ubuntu 24.04 LTS VPS, cloud-hosted). Note: The deployment was a proof-of-concept public demonstration interface and not a licensed banking institution. Transactions represent API submissions by members of the public and by the authors during testing.
Data accessibility	Repository name: Zenodo Data identification number (DOI): 10.5281/zenodo.20030064 Direct URL: https://doi.org/10.5281/zenodo.20030064 Dataset file: fraud_tests_export_20260501_080333.csv Licence: Creative Commons Attribution 4.0 International (CC BY 4.0) Access: Open access, publicly available immediately, no embargo. Zenodo is the primary and authoritative repository for this dataset. A README file containing full data documentation, column definitions, label rules, threshold logic, missing value analysis, duplicate check results, and daily transaction distribution is included in the Zenodo deposit alongside the CSV file. Note on repository identifiers: two Zenodo identifiers are associated with this deposit — the concept DOI (10.5281/zenodo.20030064), which always resolves to the latest version of the dataset, and a specific version DOI, which points to one fixed version. Both refer to the same dataset; the concept DOI is cited throughout this article and is recommended for reuse.
Related research article	Fatah, H.H. and Rashid, Z.N. A Hybrid CNN-LSTM Framework for Real-Time Online Banking Fraud Detection: From Offline Evaluation to Production Deployment. Manuscript submitted for publication (under review), 2026.

## 1 Value of the Data

• Publicly available fraud datasets are dominated by the ULB/Kaggle benchmark, which was collected in 2013 from European cardholders. To the best of the authors’ knowledge, this dataset is among the first openly available, deployment-derived fraud detection inference-log datasets collected from a live deep learning system operating in a cloud demonstration environment. It provides a recent alternative — collected in January 2026 — that reflects contemporary transaction patterns, expanding the temporal diversity of available fraud benchmarks and supporting relevant research in credit risk assessment, predictive modelling, and financial analytics [1,2,3].
• The data were collected in an uncontrolled proof-of-concept deployment environment rather than under controlled research conditions. The natural class imbalance (0.172% fraud rate) is preserved, transaction volumes vary day to day, and network conditions affect response latency. These characteristics make the data well-suited for evaluating fraud detection methods under realistic operational constraints, including imbalanced classification, calibration under distribution shift, and latency-constrained inference, that controlled or synthetically balanced benchmark datasets cannot replicate.
• Each record includes not only the transaction features and fraud label but also model output fields: fraud_probability, risk_level, confidence, recommendation, and response_time_ms. Researchers can use these fields to study threshold selection and confidence calibration without needing to re-run the model. In particular, the 12 confirmed false negative cases — which scored in the fraud_probability range 0.30–0.42, just below the decision threshold of 0.43 — illustrate the practical value of threshold calibration: a small downward adjustment of the threshold from 0.43 to approximately 0.35 would recover several of these missed fraud cases while modestly increasing false positives. Users should note that fraud_probability is the same model output that determined BLOCK/ALLOW labels, creating a circular dependency between model outputs and is_fraud labels for BLOCK-flagged records; this dependency should be explicitly accounted for in any downstream modelling that uses both columns simultaneously [4]. The dataset is primarily intended for benchmarking, simulation, and evaluation of fraud detection systems rather than unbiased supervised model training; researchers wishing to use it for model training should exclude the model-output columns (fraud_probability, risk_level, confidence, recommendation) from their feature sets to avoid information leakage.
• The data support reproducibility of the results reported in the associated companion research paper (Fatah and Rashid, 2026, submitted for publication). Independent researchers can apply their own models to these 56,962 transactions and compare detection rates directly against the CNN-LSTM results reported in that work without needing to replicate the full deployment infrastructure. Explainable AI and decision-intelligence frameworks [3] provide useful conceptual tools for interpreting the model output fields included in this dataset.
• Fraud detection research consistently identifies the scarcity of openly available, deployment-derived datasets as a barrier to real-world validation. This dataset partially addresses that gap for the fraud detection domain. To support reuse, a README file containing full data documentation, missing value analysis, duplicate check results, daily transaction distribution, threshold logic, and usage guidelines is included in the Zenodo deposit (DOI: 10.5281/zenodo.20030064, URL: https://doi.org/10.5281/zenodo.20030064). Users should note that the system was a proof-of-concept public demonstration interface and not a licensed banking institution; the transactions are API submissions and not real customer banking transactions. This distinction should be considered when assessing representativeness for licensed banking environments. Adversarial robustness and security considerations in digital financial systems [4] are additional dimensions that future research using this dataset may explore. The primary intended user groups for this dataset are: (a) researchers benchmarking and evaluating fraud detection algorithms under realistic class imbalance; (b) data scientists studying threshold calibration, confidence scoring, and decision-boundary behaviour in deployed ML systems; (c) engineers evaluating real-time API inference latency and system performance; and (d) educators and students seeking a recent, openly licensed fraud detection dataset with both raw features and model output annotations.

## 2 Background

This dataset was compiled to support the accompanying companion research article describing a hybrid CNN-LSTM fraud detection system deployed in a live cloud environment. Existing publicly available fraud datasets — most notably the ULB Credit Card Fraud Detection benchmark [5] — were generated under controlled conditions and do not capture the variability of a live operational deployment. The motivation for releasing this dataset was therefore twofold: to provide the research community with a reproducible benchmark for the companion paper, and to contribute a deployment-derived fraud dataset reflecting January 2026 transaction patterns.

It is important to state clearly that the deployment described in this article was a proof-of-concept system operating as a public demonstration interface hosted at model-test.online and was not a licensed banking institution. The 56,962 transactions in this dataset represent requests submitted to the REST API endpoint (POST /predict) by members of the public and by the authors during testing over the 31-day collection period. These transactions share the same PCA-transformed feature structure (V1–V28, Amount, and Time) as the ULB Credit Card Fraud Detection benchmark [5], because the deployed CNN-LSTM model was trained on the ULB benchmark and accepts inputs in that same format. The transaction inference logs are original records generated by the authors’ own deployed system. No transaction records were copied or replayed from the ULB dataset; only the feature structure (V1–V28, Amount, Time) is aligned with the ULB benchmark for model compatibility purposes. Researchers should interpret the dataset in this context when assessing its representativeness of real-world banking environments.

## 3 Data Description

The dataset consists of a single comma-separated values file named fraud_tests_export_20260501_080333.csv, containing 56,962 rows and 38 columns. Each row represents one transaction processed by the live deployment endpoint during the 31-day collection period (01–31 January 2026). The dataset contains 38 columns in total, of which 30 are input features (time_value, V1–V28, and amount) and the remaining 8 are model outputs and metadata fields (transaction_id, is_fraud, fraud_probability, risk_level, confidence, recommendation, response_time_ms, timestamp, and ip_address). Table 1 describes every column in the file. The descriptive statistics for selected numeric columns are presented below Table 2a.

**Table 1:** Column descriptions for fraud_tests_export_20260501_080333.csv (56,962 rows × 38 columns).

Column Name	Data Type	Range / Values	Description
transaction_id	String	Alphanumeric	Unique 8-character hexadecimal identifier assigned at ingestion. Not a modelling feature; included for record linkage only.
time_value	Float	0 – 172,792	Time elapsed in seconds from the first transaction in the dataset, consistent with the ULB benchmark convention.
amount	Float	0.01 – 25,691.16 (USD)	Transaction amount in US dollars (not normalised). Strongly right-skewed; median $49.88, mean $134.27.
V1 – V28	Float	Continuous	Twenty-eight PCA-transformed features. These share the same feature schema as the ULB/Kaggle Credit Card Fraud Detection benchmark. Original feature identities are not disclosed for privacy reasons. These are the primary input features for modelling. Note: engineered features referenced during model development (balanceDiff_Orig, amountToBalanceRatio_Dest) were intermediate variables computed prior to PCA transformation and are not present as separate columns in the distributed CSV. They are implicitly encoded within V1–V28 and cannot be independently recovered.
is_fraud	Integer	0 or 1	Ground truth fraud label. 1 = confirmed fraud (n = 98); 0 = legitimate or not individually reviewed (n = 56,864). IMPORTANT — Verification bias: labels are model-dependent. Only BLOCK-flagged transactions (fraud_probability > 0.43) were manually reviewed. ALLOW transactions were automatically assigned is_fraud = 0 without individual review. Any fraud case missed by the model threshold is permanently recorded as is_fraud = 0. Researchers must account for this when using is_fraud as ground truth.
fraud_probability	Float	0.000 – 1.000	Fraud probability score from the CNN-LSTM sigmoid output layer. Higher values indicate stronger fraud suspicion. Note: this field has a circular relationship with is_fraud for BLOCK-flagged records (see Limitations).
risk_level	String	LOW, MEDIUM, or HIGH	Categorical risk classification derived from fraud_probability with the following boundaries: LOW: fraud_probability < 0.30 MEDIUM: 0.30 ≤ fraud_probability ≤ 0.43 HIGH: fraud_probability > 0.43 MEDIUM-risk transactions always receive an ALLOW recommendation (fraud_probability ≤ 0.43). Only HIGH-risk transactions receive a BLOCK recommendation. There are no cases where a MEDIUM-risk transaction is BLOCKed.
confidence	Float	0.00 – 100.00	Model confidence expressed as a percentage. Calculated as: **confidence = |fraud_probability − 0.43| / 0.57 × 100** This represents the normalised absolute distance from the decision boundary (0.43). A high confidence value means the prediction is far from the threshold (either clearly legitimate or clearly fraudulent). This is a distance-based measure and not a conventional probabilistic confidence interval.
recommendation	String	BLOCK or ALLOW	Final action recommendation. BLOCK if fraud_probability > 0.43 (HIGH risk); ALLOW otherwise (LOW or MEDIUM risk). MEDIUM-risk transactions always result in ALLOW.
response_time_ms	Integer	42 – 893 ms	End-to-end API response latency in milliseconds, from request receipt to JSON response transmission. Includes model inference (∼45 ms), MySQL write (∼200–250 ms), and network overhead. This field is included to support research on real-time fraud detection systems, latency-constrained deployment, and system performance benchmarking. Mean: 372 ms, Median: 371 ms, Std Dev: 47 ms. The narrow std dev despite the wide range (42–893 ms) reflects that high-latency outliers are rare; 99% of transactions were processed below 650 ms (see Table 2b for percentile breakdown).
timestamp	DateTime	2026–01–01 to 2026–01–31	UTC datetime at which the transaction was received and processed by the API endpoint.
ip_address	String	IPv4 (anonymised)	Source IP address with last octet replaced by 0 to protect submitter privacy (e.g., 192.168.1.0).

**Table 2a:** Descriptive statistics for selected numeric columns.

Column	Count	Min	Max	Mean	Std Dev	Median	Fraud %
amount	56,962	0.01	25,691.16	134.27	312.84	49.88	0.172%
fraud_probability	56,962	0.000	1.000	0.043	0.142	0.008	—
response_time_ms	56,962	42	893	372	47	371	—
is_fraud	56,962	0	1	0.00172	0.041	0	—
confidence	56,962	0.00	100.00	11.8	19.4	4.3	—

**Table 2b:** Percentile distribution of response_time_ms (n = 56,962).

Percentile	Response Time (ms)	Percentile	Response Time (ms)
5th	≈48	75th	≈395
25th	≈350	95th	≈450
50th (Median)	371	99th	≈650
Mean	372	Maximum	893

The narrow standard deviation (47 ms) relative to the observed range (42–893 ms) reflects the rarity of high-latency events: 99% of transactions were processed below 650 ms. The isolated maximum of 893 ms represents sporadic network spikes rather than systematic delays. Latency components comprise model inference (≈45 ms), MySQL database write operations (≈200–250 ms), request parsing, and network round-trip overhead.

The dataset contains 56,864 records assigned is_fraud = 0 and 98 confirmed fraud cases (is_fraud = 1), yielding a fraud rate of 0.172% (56,864 + 98 = 56,962 total rows). This rate is identical to that reported in the ULB Credit Card Fraud Detection benchmark [5], despite the datasets originating from different time periods and collection contexts.

The distribution of transaction amounts is strongly right-skewed (mean $134.27, median $49.88, maximum $25,691.16). Fraudulent transactions showed a higher detection rate in the $0–$10 range (95.56%), consistent with account-testing behaviour documented in the fraud detection literature.

## 4 Experimental Design, Materials and Methods

### 4.1 System architecture

The data described in this article were collected automatically by a proof-of-concept fraud detection system deployed on a cloud Virtual Private Server running Ubuntu 24.04 LTS. The deployment was a public demonstration interface at model-test.online and was not a licensed banking institution. Transactions represent API submissions by members of the public and by the authors during testing; they are not real customer banking transactions from a licensed financial institution.

The system architecture comprises three principal components: a trained deep learning model, a RESTful API server, and a MySQL relational database.

The fraud detection model is a hybrid CNN-LSTM architecture trained on the ULB Credit Card Fraud Detection dataset (284,807 original transactions, expanded to approximately 6.3 million through SMOTE augmentation [6]). This oversampling ratio was selected to substantially raise the representation of the minority fraud class during training so that the model could learn discriminative fraud patterns despite the extreme class imbalance of the original data; full training and hyperparameter details are reported in the companion research article [7]. The model was implemented in TensorFlow 2.13.0 with Python 3.9 and containerised using Docker (image size 1.2 GB). The CNN branch uses Conv1D (64 filters, kernel size 3), BatchNormalisation, Dropout, and MaxPooling1D. The LSTM branch uses stacked LSTM layers (64 and 32 units respectively) with Dropout of 0.3. A fusion Dense layer (64 units, ReLU, Dropout 0.5) precedes the Sigmoid output layer. The decision threshold was calibrated to 0.43 based on threshold optimisation experiments on deployment data prior to the collection period.

The API server was implemented using Flask, exposed on port 5000, and reverse-proxied through Nginx. Three endpoints are relevant: GET /health (status check), POST /predict (single transaction inference), and POST /batch (batch inference up to 1000 transactions). All 56,962 transactions in this dataset were processed through POST /predict.

Transaction logs were written to a MySQL 8.0 database table named fraud_tests, with one row per API call. The schema is described in Table 3.

**Table 3:** Schema of the fraud_tests database table.

Column	MySQL Type	Notes
Id	INT AUTO_INCREMENT	Primary key; not included in the distributed CSV file.
transaction_id	VARCHAR(50)	8-character hexadecimal identifier, unique per transaction.
time_value	FLOAT	Seconds elapsed from first transaction in dataset.
amount	DECIMAL(15,2)	Transaction amount in USD, two decimal places.
v1 – v28	FLOAT	One column per PCA component, indexed v1 through v28.
is_fraud	TINYINT(1)	0 = legitimate or not individually reviewed; 1 = manually confirmed fraud.
fraud_probability	FLOAT	Raw sigmoid output, four decimal places.
risk_level	VARCHAR(10)	LOW, MEDIUM, or HIGH (see Table 1 for boundaries).
confidence	FLOAT	Distance-based confidence percentage (see Table 1 for formula).
recommendation	VARCHAR(10)	BLOCK (fraud_probability > 0.43) or ALLOW.
response_time_ms	INT	End-to-end API latency in milliseconds.
timestamp	DATETIME	UTC datetime of transaction receipt.
ip_address	VARCHAR(50)	Last octet anonymised to 0.

### 4.2 Data collection procedure

Data collection began on 01 January 2026 and concluded on 31 January 2026 (31 days). No sampling strategy was applied — every transaction submitted to the endpoint during this period was automatically logged. The following steps describe the pipeline for each transaction:

• A transaction request was submitted to POST /predict as a JSON payload containing the 30 input feature values (time_value, V1–V28, amount).
• The API server validated and parsed the request, then passed the feature vector to the pre-trained CNN-LSTM model.
• The model returned a fraud probability score. The server applied the calibrated threshold of 0.43 to assign a risk_level (LOW/MEDIUM/HIGH) and recommendation (BLOCK/ALLOW).
• A complete log record was written to the MySQL fraud_tests table.
• The API returned a JSON response including fraud_probability, risk_level, recommendation, and response_time_ms.

Important note on fraud label assignment and verification bias: Fraud labels were not assigned in real time. At the end of the collection period, all transactions with a BLOCK recommendation (fraud_probability > 0.43) were manually reviewed by the authors. Cases confirmed as fraudulent were assigned is_fraud = 1; all other BLOCK transactions were assigned is_fraud = 0 after review. All transactions with an ALLOW recommendation (fraud_probability 0.43 or below) were assigned is_fraud = 0 automatically, without individual review. This means the labelling process is model-dependent: any fraudulent transaction that the deployed model failed to flag (false negative) will be permanently recorded as is_fraud = 0 in this dataset. Researchers must account for this verification bias when using is_fraud as ground truth.

Identification of confirmed false negatives: The 12 confirmed false negatives in this dataset were identified through a targeted post-deployment audit conducted after the 31-day collection period. A manual review was performed on a sample of ALLOW-flagged transactions with fraud_probability scores in the MEDIUM-risk range (0.30 to 0.43), the range closest to the decision boundary where genuine fraud cases are most likely to be missed. This targeted audit identified 12 transactions that, upon closer inspection of transaction context and follow-up investigation, were determined to be fraudulent despite receiving an ALLOW recommendation. These 12 records are included in the dataset with is_fraud = 1, and their fraud_probability values lie in the range 0.30 to 0.42. The complete population of ALLOW transactions below the MEDIUM-risk threshold (fraud_probability below 0.30) was not audited; additional false negatives in that range cannot be excluded.

### 4.3 Feature description

The 28 features V1 through V28 share the same PCA-transformed structure as the ULB Credit Card Fraud Detection benchmark [5]. The original transaction attributes from which these principal components were derived are not disclosed, consistent with the data privacy approach of the ULB Machine Learning Group. The amount and time_value columns retain their original, untransformed values. The amount column is expressed in US dollars (USD).

During model development, engineered features including balanceDiff_Orig (correlation 0.38 with fraud label) and amountToBalanceRatio_Dest (correlation 0.18) were computed as intermediate steps prior to PCA transformation. These engineered features are not present as separate columns in the distributed CSV file (fraud_tests_export_20260501_080333.csv) and cannot be independently recovered from V1–V28. They are mentioned here only to document the model training context; researchers working with this dataset should work directly with the V1–V28 columns.

### 4.4 Ethical considerations

The data described here were collected through a public demonstration API interface. The deployment did not involve a licensed banking institution, real customer accounts, or personally identifiable financial data. No ethics committee approval was required under the research ethics guidelines of Sulaimani Polytechnic University (SPU Research Ethics Policy, Article 4: Exemption for non-human-subject research generating non-identifiable data through investigator-operated technical systems). The study qualifies for exemption because: (1) no human subjects were recruited or studied; (2) no personally identifiable information was collected; (3) the data were generated by the authors’ own research infrastructure and not obtained from third-party organisations or individuals.

Data-use authorisation: The system was operated by the authors at model-test.online as a research proof-of-concept. All data generated by the system were produced by the authors’ own infrastructure and are owned by the authors. No consent waiver was required as no human subject data was collected.

Privacy risk assessment: The feature columns V1–V28 are PCA-transformed and cannot be reverse-transformed to recover the original transaction attributes. IP addresses were anonymised at collection time by replacing the last octet with zero. Transaction amounts do not include account identifiers, cardholder names, or card numbers. The dataset presents a low privacy risk and does not contain information that could identify individual persons.

## Limitations

Several limitations of this dataset should be noted by potential users.

First, the collection period is restricted to 31 days in January 2026. Seasonal patterns in fraud behaviour occurring at other times of year are not represented, and the dataset cannot be assumed to reflect annual fraud trends.

Second, the dataset was collected through a proof-of-concept public demonstration deployment and not through a licensed banking institution. Transactions are API submissions by the public and by the authors during testing; they are not real customer banking transactions. Transaction volumes (∼1900 per day) are lower than those of a full-scale banking system. Generalisability to licensed banking environments should not be assumed without further validation.

Third, ground truth fraud labels are model-dependent. Only BLOCK-flagged transactions (fraud_probability > 0.43) were manually reviewed; ALLOW transactions were automatically labelled as legitimate without individual review. This verification bias means that fraudulent transactions with fraud_probability ≤ 0.43 are permanently recorded as is_fraud = 0. A targeted audit of MEDIUM-risk transactions (0.30–0.43) identified 12 false negatives, but false negatives in the LOW-risk range (fraud_probability < 0.30) cannot be excluded. Additionally, the fraud_probability column has a circular dependency with is_fraud for BLOCK-flagged records, since the same model output was used to determine both the label assignment and the stored probability score.

Fourth, the features V1–V28 are anonymised PCA components whose original identities are not disclosed, limiting the degree to which domain knowledge can be applied to feature engineering. The engineered features used during model development (balanceDiff_Orig, amountToBalanceRatio_Dest) are not available as separate columns in the distributed file.

## Ethics Statement

The authors confirm that the work described in this article does not involve human subjects, animal experiments, or data collected from social media platforms. The dataset was generated through automated API logging of transactions submitted to a public proof-of-concept demonstration interface. No personally identifiable information was collected or retained. No ethics committee approval was required under the guidelines of Sulaimani Polytechnic University applicable to this type of research. The authors have read and follow the ethical requirements for publication in Data in Brief.

## CRediT Author Statement

**Hemn Hashim Fatah:** Conceptualisation, Data curation, Formal analysis, Investigation, Methodology, Software, Validation, Visualisation, Writing — original draft, Writing — review and editing.

**Zryan Najat Rashid:** Conceptualisation, Funding acquisition, Project administration, Resources, Supervision, Writing — review and editing.

## Data Availability

ZenodoDeployment-Derived Online Banking Fraud Detection Inference-Log Dataset (Original data)
